# Opioid Consumption After Upper Extremity Surgery: A Systematic Review

**DOI:** 10.1177/15589447231160211

**Published:** 2023-03-23

**Authors:** Minh N. Q. Huynh, Morgan Yuan, Lucas Gallo, Oluwatobi R. Olaiya, Jouseph Barkho, Matthew McRae

**Affiliations:** 1 McMaster University, Hamilton, Ontario, Canada; 2 University of Toronto, Ontario, Canada

**Keywords:** hand, anatomy, shoulder, pain management, specialty, surgery, psychosocial, research and health outcomes

## Abstract

There is currently an overprescription of opioids, which may result in abuse and diversion of narcotics. The aim of this systematic review was to investigate opioid prescription practices and consumption by patients after upper extremity surgery. This review was registered a priori on Open Science Framework (osf.io/6u5ny) and adhered to the Preferred Reporting Items for Systematic Reviews and Meta-Analyses guidelines. A search strategy was performed using MEDLINE, Embase, PubMed, and Cochrane Central Register of Controlled Trials databases (from their inception to October 17, 2021). Prospective studies investigating opioid consumption of patients aged 18 years or older undergoing upper extremity surgeries were included. The Risk of Bias in Nonrandomized Studies of Interventions and Risk of Bias 2.0 tools were used for quality assessment. In total, 21 articles met the inclusion criteria, including 7 randomized controlled trials and 14 prospective cohort studies. This represented 4195 patients who underwent upper extremity surgery. Most patients took less than half of the prescribed opioids. The percentage of opioids consumed ranged from 11% to 77%. There was moderate to severe risk of bias among the included studies. This review demonstrated that there is routinely excessive opioid prescription relative to consumption after upper limb surgery. Additional randomized trials are warranted, particularly with standardized reporting of opioid consumption and assessment of patient-reported outcomes.

## Introduction

In the 1990s, there was a paradigm shift to prioritize pain control.^1^ This was pioneered by Dr James Campell, who identified pain as the fifth vital sign.^2^ At that time, research highlighted the undertreatment of pain, subsequently promoting the use of opioid medications. However, the addictive properties and side effects were minimized by publications, which resulted in unforeseen consequences.^3^ For instance, a 1980 study by Porter et al that was published in *The New England Journal of Medicine* reported a very low incidence (4 of 11 882 patients) of narcotic addiction after narcotic use in hospital and has since garnered more than 1500 citations on Google Scholar.^4^ In the decades following, the rate of postoperative opioid prescriptions rose without regard to the risks of opioids due to misinformation from pharmaceutical companies and legal repercussions from undertreating pain.^5^ The opioid epidemic has had profound effects from the patient, societal, and physician perspective. In 2010, more than 5 million Americans had used opioid medications for nonmedical purposes within the past month, resulting in significant emergency department visits and billions of dollars lost in work productivity.^5^ A study by Birnbaum et al found the estimated societal costs in the United States of prescription opioid abuse to be $55.7 billion in 2007.^6^ Moreover, multiple studies demonstrate that physicians continue to routinely overprescribe opioid analgesics despite the fact that most prescription opioids remain unused.^5,7,8^

Opioids have been routinely prescribed postoperatively for upper extremity surgeries. However, it is controversial whether the benefits of routine opioid prescription after upper extremity surgery outweigh the risks.^4^ Therefore, the authors would like to investigate opioid consumption by patients after upper extremity surgery.

## Methods

### Search Strategy and Study Selection

**Figure 1.**
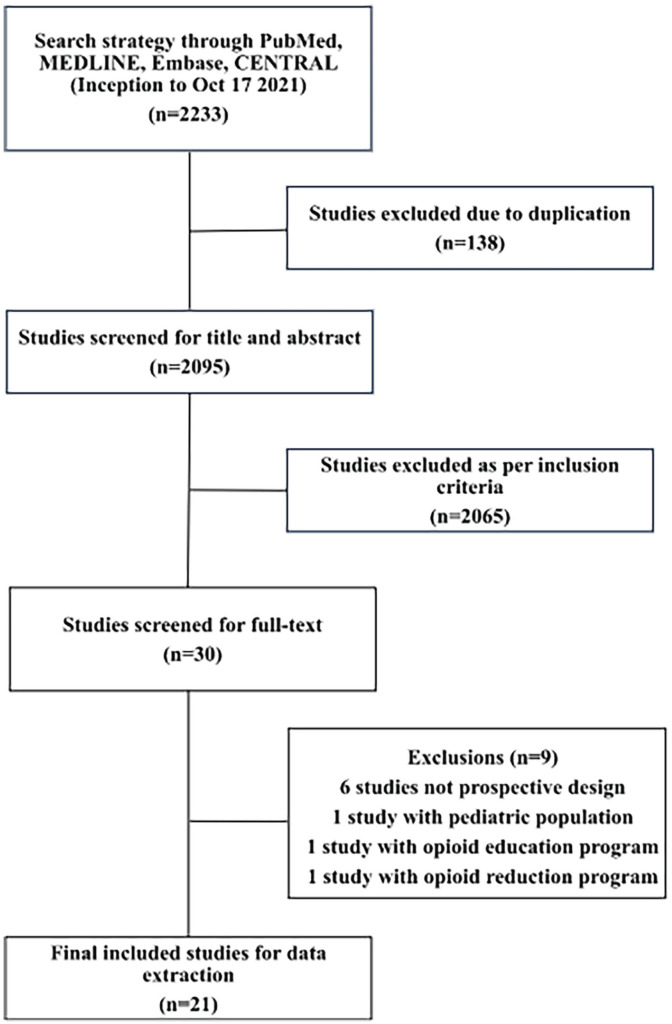
Preferred Reporting Items for Systematic Reviews and Meta-Analyses diagram of included studies.

This review was reported in concordance with the Preferred Reporting Items for Systematic Reviews and Meta-Analyses guidelines (Figure 1) and was prospectively registered with the Open Science Framework (osf.io/6u5ny). A literature search (Supplemental Material) was developed and performed using PubMed, Ovid MEDLINE, Embase, and Cochrane Central Register of Controlled Trials (CENTRAL) from their respective inception to October 17, 2021.The articles were uploaded to Rayyan^9^ for screening. The articles were then screened independently and in duplicate, at both title and abstract and full-text levels, by 2 reviewers.

Articles were included if they featured the following inclusion criteria:

1. Prospective study design including randomized controlled trials (RCTs), nonrandomized controlled trials, or observational cohort studies;
2. Patients undergoing upper extremity surgery;
3. Patients who are older than 18 years;
4. Documentation of opioid prescription or consumption;
5. English as the language of publication.

Articles were excluded if they were not primary research articles (ie, abstracts, conference proceedings, etc), if the data could not be extracted, if they were retrospective study designs or case report studies, if the intervention was a protocol for opioid reduction program, or if the intervention involved an opioid education program.

The primary objective of the study was to determine prescription opioid consumption after upper extremity surgery. Primary outcomes of interest were morphine milligram equivalents (MMEs) of opioid prescribed and MMEs consumed by the patient. The secondary outcomes of interest included opioid prescription and consumption by location of surgery, adverse effects, and pain scores.

### Data Extraction and Quality Assessment

Data from the included articles were independently extracted in duplicate by 2 reviewers using a predefined, standardized data collection instrument. Any disagreements were resolved by discussion to reach a consensus. If a consensus could not be obtained, any conflicts were resolved by the corresponding author. Extracted data included demographic information (age, sex, comorbidities, etc), study design, opioid prescribing and consumption patterns which were converted to MMEs, type of surgical anesthetic, type of surgery, location of surgery, and follow-up time. The MMEs of some commonly prescribed opioids include codeine (0.15), hydrocodone (1), hydromorphone (4), morphine (1), oxycodone (1.5), and oxymorphone (3).

Two reviewers independently assessed the studies for risk of bias and applicability of the study methodology. For each article, the risk of bias assessment was performed using the Risk of Bias in Nonrandomized Studies of Interventions (ROBINS-I) tool ^10^ for nonrandomized studies or Risk of Bias (RoB) 2.0 for RCTs.^11^

### Statistical Analysis

Descriptive statistics were reported for patient demographic information and study characteristics. Opioid prescriptions and consumption were converted to MMEs to standardize values. Due to the inconsistency of outcome reporting and missing data, a meta-analysis could not be performed. All data analyses were performed in R statistical software (version 3.6.1).^4^ Inter-rater reliability for screening at the title and abstract level was calculated using Cohen’s κ score. Values of 0.01 to 0.20, 0.21 to 0.40, 0.41 to 0.60, 0.61 to 0.80, and 0.81 to 0.99 were considered slight, fair, moderate, substantial, and excellent agreement, respectively.^12^

## Results

The initial literature search identified 2233 studies. After 138 studies were removed as duplicates, 2095 studies were screened at the title and abstract level. Subsequently, 30 studies were screened for inclusion at the full-text level. After applying the inclusion criteria, 21 studies^8,13-32^ were included for the final analysis, including 7 RCTs and 14 prospective studies. Figure 1 outlines the screening process and the reasons for exclusion at the full-text level. Cohen’s κ was calculated to be 0.803, which represents excellent agreement between reviewers.

### Study Characteristics

The 21 included studies contributed a total of 4195 patients who underwent upper extremity surgery, of which 3826 patients received prescriptions for opioid analgesics. Details of included studies can be found in Table 1 for included RCTs and Table 2 for included prospective observational studies. All studies were performed in North America, either in the United States (n = 20) or in Canada (n = 1). The mean age of patients ranged from 48 to 65 years. There were 1780 male patients and 2236 female patients who underwent upper extremity surgery, although 2 studies did not report the sex distribution of patients.^15,20^ There was heterogeneity in the type of anesthesia received by patients, with 44% receiving general anesthesia/monitored anesthetic care/local anesthesia with sedation (n = 1834), 27% receiving local anesthesia (n = 1152), 12% receiving regional anesthesia (n = 492), and 17% did not specify the anesthesia type (n = 717). Most surgeries were performed on the hand and wrist (75%, n = 3156), followed by shoulder (12%, n = 490), elbow (6%, n = 263), and forearm (1%, n = 41). Two studies did not use the same anatomical distribution groups.^15,21^ The follow-up period of included studies ranged from 3 to 90 days.

**Table 1.** Study Characteristics of Included Randomized Controlled Trials.^13-19^

Study	Subgroup	Country	Anatomical region	Procedure	No. of patients	Age (SD)	Sex (M/F)	Outcomes	Opioid prescribed	Mean MMEs of opioids prescribed (SD)	Mean MMEs of opioids consumed (SD)
Alter et al^13^	Marcaine injection	USA	Forearm	Distal radius fracture open reduction and Internal fixation	21	57 (15)	4/17	Daily opioids, pain, adverse reaction	20 pills of OXY/ACE (5/325 mg); HYD/ACE (5/300 mg); or ACE/COD	90-150^a^	53.6
	Exparel injection				20	63 (15)	4/16				45.7
Gaddis et al^14^	10 HYD/ACE	USA	Hand	Minor hand surgery	95	51.3 (16-69)^a^	29/66	Opioid use, continued opioid use, pain	HYD/ACE (5/325 mg)	59 (28.5)	32 (21.5)
	30 HYD/ACE				79		23/56		HYD/ACE (5/325 mg)	161 (39)	59.5 (70)
Harrison et al^15^	Control	USA	Wrist/forearm	Carpometacarpal arthroplasty or distal radius fracture open reduction and internal fixation	18	N/A	N/A	Medication use, numeric pain score, adverse effects	Percocet (OXY/ACE [5/325]) 5-10 mg	N/A	183
	OXY				20				1. OXY 0-15 mg 2. ACE 650 mg every 6 h, scheduled.	N/A	183
	OxyContin				17				1. OXY 0-15 mg. 2. ACE 650 mg every 6 h, scheduled. 3. OxyContin (OXY sustained release) 10 mg twice a day, scheduled.	N/A	183
	Ketorolac				24				1. OXY 0-15 mg 2. ACE 650 mg every 6 h, scheduled. 3. Toradol (ketorolac) 10 mg every 6 h, scheduled.	N/A	144.75
Ilyas et al^16^	Endoscopic OXY	USA	Hand	Endoscopic CTR	19	59 (47-73)^a^	11/8	Pain, pills consumed, adverse effects	OXY 5 mg × 10 tablets	75	21.75
	Endoscopic IBU			Endoscopic CTR	16	61 (45-87)^a^	6/10		IBU 600 mg (10 capsules)	0	0
	Endoscopic ACE			Endoscopic CTR	24	60 (32-74)^a^	13/11		ACE 500 mg (10 capsules)	0	0
	Open OXY			Open CTR	18	61 (29-84)^a^	6/12		OXY 5 mg × 10 tablets	75	27.75
	Open IBU			Open CTR	18	64 (19-88)^a^	11/7		IBU 600 mg (10 capsules)	0	0
	Open ACE			Open CTR	10	66 (45-88)^a^	5/5		ACE 500 mg (10 capsules)	0	0
Ilyas et al^17^	OXY	USA	Hand	CTR or trigger finger release	62	60 (12.1)	26/36	Pain, pills consumed, adverse effects	OXY 5 mg (10 capsules)	75	Open CTR (30.75), endoscopic CTR (21.75), open TFR (20.25)
	IBU				64		26/38		IBU 600 mg (10 capsules)	0	0
	ACE				62		28/34		ACE 500 mg (10 capsules)	0	0
Tangtiphaibootana et al^18^	IBU	USA	Shoulder	Arthroscopic repair of rotator cuff	51	57.7 (10.8)	29/22	Opioid consumption, pain (VAS), shoulder ROM, DASH, and ASES score	HYD/ACE (10/325 mg) × 60 pills	600 (0)	168.3 (96)
	Placebo			Arthroscopic repair of rotator cuff	50	56.9 (13.8)	29/21		HYD/ACE (10/325 mg) × 60 pills	600 (0)	210.9 (104)
Weinheimer et al^19^	Opioid	USA	Hand	Hand surgery	30	53 (18-75)	13/17	Opioid consumption, pain (VAS), rescue opioids at 1 wk	ACE/HYD (325/5 mg)	210 (0)	50 (40)
	Nonopioid			Hand surgery	30	52 (18-86)	12/18		ACE/IBU (500/400 mg)	0 (0)	0

*Note.* MMEs = mean morphine equivalents; OXY = oxycodone; ACE = acetaminophen; HYD = hydrocodone; COD = codeine; IBU = ibuprofen; CTR = carpal tunnel release; VAS = Visual Analog Scale; ROM = range of motion; DASH = Disabilities of the Arm, Shoulder, and Hand; TFR = total femoral replacement; ASES = American Shoulder and Elbow Surgeons Standardized Shoulder Assessment Form.

a Range.

**Table 2.** Study Characteristics of Included Prospective Observational Studies.^8,20-32^

Study	Subgroup	Country	Anatomical region	Procedure	No. of patients	Age (SD)	Sex (M/F)	Outcomes	Opioid prescribed	Mean MMEs of opioids prescribed (SD)	Mean MMEs of opioids consumed (SD)
Chapman et al^8^	Percocet	USA	Hand	Carpal tunnel release	277	64.6 (22-90)^a^	123/154	Variables associated with opioid consumption	Percocet (assume 5 mg OXY)	157.5	33
	Tylenol 3								Tylenol 3 (assume 30 mg COD)	94.5	18.45
	Vicodin								Vicodin (assume 5 mg HYD)	105	19.5
	HYD								HYD (assume 5 mg)	105	23
Hozack et al^20^	Cubital tunnel	USA	Elbow	Cubital tunnel release	100	N/A	N/A	Opioid consumption, QuickDASH score	Percocet (5 mg OXY/325 mg ACE) or an OXY 5 mg equivalent, Vicodin (5 mg HYD/325 mg ACE) or an HYD 5 mg equivalent, and Tylenol 3 (30 mg COD and 325 mg ACE)	N/A	50 (0-300)^a^
Kim et al^21^	Upper extremity surgery	USA	Upper extremity	All upper extremity surgery	1416	56 (18-93)^a^	639/777	Opioid consumption	Percocet (OXY and ACE) or an OXY 5 mg equivalent, Vicodin (ACE and HYD) or an HYD 5 mg equivalent, and Tylenol 3 (ACE and COD) with 30 mg of COD.	108-180 (0-110)^a^	36.45-60.75 (0-90)^a^
Kumar et al^22^	Shoulder	USA	Shoulder	Rotator cuff repair	100	48 (14)	74/26	Opioid consumption, patient satisfaction, education	OXY/ACE (5/325 mg) (64 patients), OXY/ACE (10/325 mg) (28 patients), HYD/ACE (5/325 mg) (4 patients), HYD 2 mg (3 patients), COD/ACE (1 patient)	248.85-829.5^a^	20.1, median 13 (IQR: 0-32)
Miller et al^23^	WALANT	USA	Hand	Carpal tunnel release and trigger finger release	181	64.4 (22-89)^a^	83/98	Opioid consumption	Vicodin, Tylenol 3, and Percocet	90-150^a^	17.325-28.875^a^ (30.15-50.25)^a^
	Monitored anesthesia care				235	63.7 (29-89)^a^	99/136		Vicodin, Tylenol 3, and Percocet	90-150^a^	17.775-29.625^a^ (25.65-42.75)^a^
Miller et al^24^	Opioid	USA	Hand	Carpal tunnel release (open or endoscopic)	159	59.82	70/89	Opioid consumption	HYD, 5 mg (Vicodin, Lortab, Norco); COD, 30 mg (Tylenol 3); and OXY, 5 mg (Percocet).	90-150^a^	22.05-36.75^a^
	Tramadol				110	58.85	47/63		Tramadol (assume 37.5 mg)	0	0
Miller et al^25^	Peripheral nerve block	USA	Hand	Thumb basal joint arthroplasty	27	64	7/20	Opioid consumption and pain (VAS)	Percocet (5 mg OXY and 325 mg ACE), Vicodin (5 mg HYD and 300 mg ACE), or Tylenol 3 (30 mg COD and 325 mg ACE).	N/A	76.5-127.5^a^
	Local anesthesia with bupivacaine				23	60	4/19			N/A	85.5-142.5^a^
	Local anesthesia with liposomal bupivacaine				28	62	8/20			N/A	49.5-82.5^a^
O’Neil et al^26^	Distal radius fracture	USA	Forearm	Distal radius fracture open reduction and internal fixation	98	58 (13-92)^a^	19/79	Opioid consumption	OXY/ACE or OXY equivalent, HYD/ACE or HYD equivalent, and ACE/COD.	N/A	58.5 (0-280)^a^
Patel et al^27^	Arthoscopic	USA	Shoulder	Arthroscopic repair of rotator cuff	41	59 (8.98)	33/8	Opioid consumption	Not specified, majority were 20 pills of 5 mg OXY and 20 pills of 10 mg Toradol	165.8	128.25 (0-450)^a^
	Arthroplasty			Total shoulder arthroplasty	14	60.57 (11.38)	10/4			165	87.8 (7.5-315)^a^
	Reverse			Reverse shoulder arthroplasty	16	59.94 (11.4)	9/7			162.8	56.3 (0-255)^a^
	Other			Distal clavicle dissection or labral surgery	9	56.33 (10.31)	7/2			158.3	108.8 (0-275)^a^
Peters et al^28^	Paracetamol with tramadol	Canada	Hand	Carpal tunnel release (open)	13	57 (18-88)^a^	17/32	Opioid consumption	Paracetamol with tramadol (37.5 mg)	0	0
	Paracetamol with COD				36				Paracetamol with COD (30 mg COD)	153	45
Rodgers et al^29^	Hard tissue	USA	Upper extremity	Upper extremity surgery	58	54 (12.8)	83/167	Opioid consumption, satisfaction with pain control, adverse events	HYD, OXY, propoxyphene, COD, unknown, ACE, IBU, or propoxyphene, and HYD	135-225^a^	63-105^a^
	Soft tissue				191					135-225^a^	40.5-67.5^a^
Staples et al^30^	In situ decompression	USA	Elbow	In situ decompression for cubital tunnel surgery	47	51 (12)	23/24	Opioid consumption, pain (VAS), patient-reported disability (Levine-Katz, Patient-Rated Elbow Evaluation)	N/A	N/A	Median 43 (IQR: 143)
	Ulnar nerve transposition			Ulnar nerve transpositions for cubital tunnel surgery	78	49 (13)	42/36		N/A	N/A	Median 225 (IQR: 384)
Stepan et al^31^	Control	USA	Hand	Hand surgery	54	54.9 (14)	23/31	Opioid consumption and pain (VAS)	14 capsules 200 mg celecoxib + 30 pills of 5 mg/325 mg HYD/ACE	150	Median 20 (0-250)^a^
	Celecoxib				33	48.6 (16)	12/21		30 pills of 5 mg/325 mg HYD/ACE	150	Median 30 (0-330)^a^
Williams et al^32^	Open	USA	Shoulder	Open repair of rotator cuff	52	60.1 (95% CI, 57.3-62.9)	37/15	Opioid consumption and pain (VAS)	OXY/ACE (5/325 mg)	N/A	380.4 (95% CI, 249.1-511.7)
	Arthroscopic			Arthroscopic repair of rotator cuff	50	57.6 (95% CI, 54.9-60.3)	36/14		OXY/ACE (5/325 mg)	N/A	327.8 (95% CI, 239.8-415.8)

*Note.* MMEs = mean morphine equivalents; HYD = hydrocodone; OXY = oxycodone; COD = codeine; ACE = acetaminophen; WALANT = wide-awake local anesthesia no tourniquet; IBU = ibuprofen; VAS = Visual Analog Scale; CI = confidence interval; IQR = interquartile range; QuickDASH = shortened Disabilities of the Arm, Shoulder, and Hand.

a Range.

### Outpatient Opioid Use

Fifteen of the included studies^8,13,14,16-19,21-24,27-29,31^ provided details on mean or range of opioids prescribed, whereas all included studies provided details on mean or range of opioids consumed. There was heterogeneity in the opioids prescribed including Tylenol 3 (or codeine/acetaminophen equivalent), tramadol, paracetamol/tramadol, paracetamol/codeine, propoxyphene, propoxyphene/hydrocodone, Vicodin (or hydrocodone/paracetamol equivalent), hydrocodone, hydrocodone/acetaminophen, oxycodone, or Percocet (or oxycodone/acetaminophen equivalent). The mean MMEs prescribed ranged from 59 to 600. The mean MMEs consumed ranged from 18.45 to 380.4.

Three studies^15,21,29^ did not stratify opioid consumption by anatomical region. Ten studies^8,14,16,17,23-25,28,31,32^ described opioid consumption in hand/wrist surgeries. The range of mean MME prescribed was 0 to 210 and consumed was 0 to 59.5. Two studies^13,26^ described opioid consumption in forearm surgeries. The range of mean MME prescribed was 90 to 150 and consumed was 45.7 to 58.5. Two studies^20,30^ described opioid consumption in elbow surgeries, but neither provided details on MMEs of opioid prescribed. The range of mean MME consumed was 0 to 59.5. Four studies^18,22,27,32^ described opioid consumption in shoulder surgeries. The range of mean MME prescribed was 108 to 600 and consumed was 55.9 to 380.4.

### Adverse Events

Five studies^13,15-17,25^ reported on adverse events, all of which were RCTs. Adverse events were present in 11% to 29% of patients. This was most commonly in the form of nausea, but in some cases also including pruritus, constipation, dizziness, and lack of energy. No major adverse or allergic reactions that required hospitalization were reported.

### Pain Scores

Three studies^14,22,29^ reported patient satisfaction with pain control, with 70% to 92% of patients reporting adequate control with the prescribed opioid analgesics. Ten studies^13,15-19,25,30-32^ reported postoperative pain scores. The average postoperative pain score within 1 week of the surgery ranged from 2.1 to 5.4. This was most commonly an average Visual Analog Scale pain score across the first 5 postoperative days. Three of these studies^18,30,32^ followed up patients for pain scores for longer durations, ranging from 6 weeks to 1 year.

### Risk of Bias Assessment

**Figure 2.**
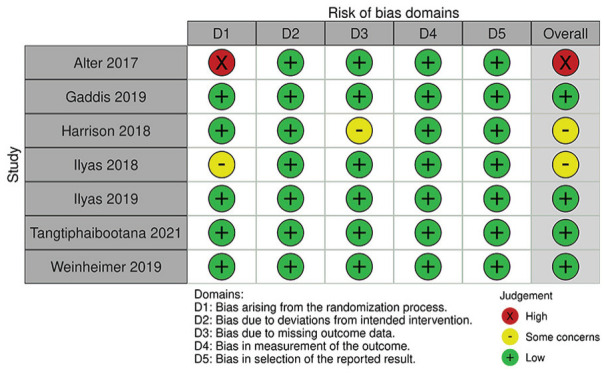
Risk of bias assessment in randomized controlled trials.

**Figure 3.**
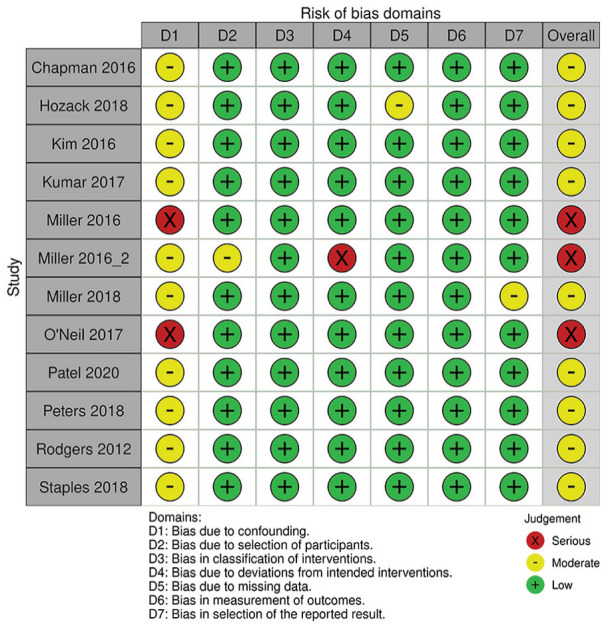
Risk of bias assessment in prospective observational studies.

A detailed breakdown of the risk of bias assessments is provided in Figure 2 for RCTs and Figure 3 for prospective observational studies. Among 7 RCTs, 4 were found to have low risk of bias, but 2 had some concerns and 1 was rated at high risk of bias due to date of birth randomization. Among the 14 prospective observational studies, 8 had low risk of bias and 6 had moderate risk of bias.

## Discussion

To the authors’ knowledge, this is the first systematic review to investigate the opioid consumption in patients undergoing upper extremity surgery. This study evaluated 7 RCTs and 14 prospective observational studies, which represented 4195 patients. Overall, the randomized and observational study evidence suggests that there is an overprescription of opioids among patients in this cohort. Our current review found that despite significant heterogeneity in the reporting opioid prescription patterns, most patients took less than half of the prescribed opioids. The percentage of opioids consumed ranged from 11% to 77%.

Among the included studies, risk of bias assessment using the RoB 2.0 and ROBINS-I tool demonstrated moderate risk of bias within randomized and nonrandomized studies. For example, there were “some concerns” and “high” risk of bias among RCTs in missing outcome data and randomization process, respectively (Figure 2). Specifically, 1 published RCT^17^ did not provide sufficient evidence that random allocation of participants had taken place. Another RCT^13^ allocated patients by date of birth, which was not an adequate means of randomizing patients. Similarly, there were “moderate” and “serious” concerns of risk of bias in observational studies. Particularly, all primary studies did not account for all important confounding variables, such as previous opioid use. Other important confounding variables include demographic factors, type of surgery (particularly by anatomical distribution or surgery type), and type of anesthetic. Future studies investigating opioid use should use appropriate methods to randomize patients in RCTs and control for confounding variables a priori in observational studies to ensure results are comparable across study groups.

The findings of this study are in keeping with other systematic reviews investigating the prescription of opioids after surgery. Feinberg et al^33^ conducted a systematic review to identify patients prescribed opioids for analgesia after various elective surgeries (eg, C-sections and common outpatient general surgery procedures). They found that most patients consumed 15 or less opioid pills, which is compared with the routine practice of prescribing 30 or more opioid pills.^29^ The percentages of opioids consumed ranged from 11% to 90.1% in their study. In conjunction with this study, it is estimated patients are prescribed nearly double the opioid pills necessary to achieve analgesia. Similarly, a systematic review by Bicket et al^7^ investigated opioid prescriptions after surgical procedures (eg, thoracic surgery and dermatology) in both inpatient and outpatient settings. They found that the number of opioid pills unused postoperatively ranged from 42% to 71% of pills dispensed. Although this study had limited evidence with inclusion of only 6 studies, the findings are in keeping with this study that identified similar overprescription patterns in a larger cohort of patients undergoing upper extremity surgery.

The overprescription of opioid pills for analgesia is concerning, given the potential harms. Despite difficulty in ascertaining whether patients develop opioid dependence after opioid prescriptions, the opioid crisis continues to worsen with 1.9 million people in the United States meeting criteria for opioid abuse or dependence in 2013.^34^ Therefore, it is crucial that physicians evaluate prescription patterns for opioids and consider reductions. Although not our primary objective, this systematic review identified patients experiencing adverse events in 11% to 29% of patients. It has been reported that 10% to 30% of patients achieve poor pain control outcomes due to intolerable side effects from opioids, including sedation and nausea/vomiting.^35^ In addition, physicians should consider secondary harms from unused opioids including opioid diversion. Previous studies have demonstrated that increased opioid diversion is correlated with deaths from opioids.^36,37^ Current guidelines suggest that procedure-specific approaches to opioid prescriptions should be a focus of future research^38^ and that nonopioid adjuncts should be considered for analgesia. For instance, Overton et al developed consensus ranges for 20 surgical procedures with an expert panel through a 3-step modified Delphi method.^38^ Such studies would undoubtedly better advise surgeons on outpatient analgesia. In addition, some procedures such as those of the hand may not even require opioid analgesics. For example, Ilyas et al^17^ performed an RCT investigating analgesia for hand surgeries using oxycodone, ibuprofen, and acetaminophen. They did not find significant differences in pain scores between groups, and subgroup analysis found significantly higher pain among patients undergoing open carpal tunnel release and taking oxycodone. This study demonstrates that opioid analgesics are not always necessary and that nonopioid analgesia should be considered in solidarity or in conjunction with opioids. Further research on procedure-specific analgesia guidelines may help streamline physician decision-making.

There were several limitations that were identified in this study. Gray literature was not included in the search strategy, which reduces the available evidence and may introduce publication bias. There was also a lack of standardized reporting in opioids prescribed or taken across several of the included studies. This heterogeneity in data made a meta-analysis unfeasible. Future studies should use standardized approaches in reporting such as MMEs to provide detailed evidence of opioid consumption. Geographically, all the included studies were conducted in North America, which limits the generalizability of findings as the prescription patterns of other health care systems may differ. In addition, the patient satisfaction surveys used in several studies may also influence prescription patterns as physicians can be pressured to prescribe more opioids for analgesia. Furthermore, publication lag may also introduce bias as studies are likely published several years after data collection and are not reflective of current practices. Finally, there was a lack of reporting of patient-reported outcomes, with only 10 studies assessing pain and 3 studies assessing patient satisfaction with analgesia. Patient-reported outcomes are pivotal in opioid research as they provide subjective evidence to compare different means of analgesia.

In conclusion, this review demonstrated an excessive opioid prescription relative to consumption in patients undergoing upper extremity surgery. Surgeons are advised to consider reduction of up to 50% in opioid prescriptions postoperatively from upper extremity surgery. Going forward, additional randomized trials are necessary to evaluate the differences in analgesia using opioids and other nonopioid adjuncts pertaining to specific surgical procedures. Standardized reporting of outcomes, such as opioid consumption and patient-reported outcomes, will help evaluate this research question. Such studies can advise researchers to develop procedure-specific guidelines for analgesic prescription and will help reduce overprescription of opioids.

## Supplemental Material

sj-docx-1-han-10.1177_15589447231160211 – Supplemental material for Opioid Consumption After Upper Extremity Surgery: A Systematic Review Supplemental material, sj-docx-1-han-10.1177_15589447231160211 for Opioid Consumption After Upper Extremity Surgery: A Systematic Review by Minh N. Q. Huynh, Morgan Yuan, Lucas Gallo, Oluwatobi R. Olaiya, Jouseph Barkho and Matthew McRae in HAND
