# Supplementary material for: Opioid Consumption After Upper Extremity Surgery: A Systematic Review
Source: Hand (N Y). 2023 Mar 23;19(6):1002–11. doi: 10.1177/15589447231160211 (PMC11342701; doi:10.1177/15589447231160211)
Supplement: sj-docx-1-han-10.1177_15589447231160211 – Supplemental material for Opioid Consumption After Upper Extremity Surgery: A Systematic Review [file sj-docx-1-han-10.1177_15589447231160211.docx]

**Appendix A. Search strategy**

This appendix provides readers with the search strategy used to identify possible studies for inclusion in our study.

### **Pubmed**

("Upper Extremity"[Mesh] OR "Bones of Upper Extremity"[Mesh] OR "Elbow Joint"[Mesh] OR "Hand Joints"[Mesh] OR "Shoulder Joint"[Mesh] OR "Wrist Joint"[Mesh] OR "Arm Injuries"[Mesh] OR "Brachial Plexus"[Mesh])

OR

("Carpal Tunnel Syndrome"[Mesh] OR "Trigger Finger Disorder"[Mesh] OR "Ulnar Nerve Compression Syndromes"[Mesh] OR "De Quervain Disease"[Mesh] OR "Dupuytren Contracture"[Mesh] OR "Surgery, Plastic"[Mesh] OR "Radial Neuropathy"[Mesh])

OR

(

**("Soft Tissue Injuries"[Mesh] OR "Fractures, Bone"[Mesh] OR "Arthrodesis"[Mesh] OR "Tendon Transfer"[Mesh] OR "Tendon Injuries"[Mesh] OR “Neoplasms”[Mesh] OR "Amputation"[Mesh] OR "Arthroplasty"[Mesh] OR "Arthroscopy"[Mesh] OR "Fracture Fixation"[Mesh] OR "Osteotomy"[Mesh] OR "Tenodesis"[Mesh] OR "Crush Injuries"[Mesh] OR "Peripheral Nerve Injuries"[Mesh])**

AND

("Upper Extremity"[Mesh] OR "Bones of Upper Extremity"[Mesh] OR "Elbow Joint"[Mesh] OR "Hand Joints"[Mesh] OR "Shoulder Joint"[Mesh] OR "Wrist Joint"[Mesh] OR "Arm Injuries"[Mesh] OR "Brachial Plexus"[Mesh])

)

AND

((narcotics[MeSH Terms]) or opioid*)

### **MEDLINE+ COCHRANE**

exp Narcotics/ or opioid*.mp. or narcotic*.mp.

AND

Exp Surgery, Plastic/

OR

exp Upper Extremity/ or exp elbow joint/ or exp hand joints/ or exp shoulder joint/ or exp arm bones/ or exp hand bones/ or exp shoulder injuries/ or exp Arm Injuries/ or exp hand injuries/ or exp Brachial Plexus/

OR

exp Dupuytren Contracture/

OR (w above for each)

exp median neuropathy/ or exp radial neuropathy/ or exp ulnar neuropathies/ or exp Tendon Entrapment/ or exp Fractures, Bone/ or exp fractures, cartilage/ or exp Soft Tissue Injuries/ or exp arthrodesis/ or exp arthroplasty/ or exp arthroscopy/ or exp fracture fixation/ or exp osteotomy/ or exp tendon transfer/ or exp tenodesis/ or exp crush injuries/ or tendon injuries/ or exp Amputation/ or exp Neoplasms/

### **EMBASE**

exp narcotic agent/ or opioid*.mp. or narcotic*.mp.

AND

exp hand reconstruction/ or exp microvascular surgery/ or exp nerve reconstruction/ or exp reimplantation/ or exp skin surgery/ or exp surgical flaps/ or exp tendon reconstruction/

OR

Exp upper limb/ or exp elbow/ or exp radioulnar joint/ or exp brachial plexus/ or exp "bones of the arm and hand"/

OR

exp Dupuytren contracture/ or exp carpal tunnel syndrome/ or exp cubital tunnel syndrome/ or exp radial neuropathy/

OR

exp arm disease/ or exp nerve injury/ or exp amputation/ or exp bone resection/ or exp "closed reduction (procedure)"/ or exp fracture treatment/ or exp hand surgery/ or exp joint surgery/ or exp "open reduction (procedure)"/ or exp osteotomy/ or exp tendon surgery/ or exp fracture/ or exp soft tissue injury/ or exp arthrodesis/ or exp tenodesis/ or exp crush trauma/ or exp neoplasm/
